# *In silico* validation of potent phytochemical orientin as inhibitor of SARS-CoV-2 spike and host cell receptor GRP78 binding

**DOI:** 10.1016/j.heliyon.2021.e05923

**Published:** 2021-01-11

**Authors:** Arijit Bhowmik, Souradeep Biswas, Subhadip Hajra, Prosenjit Saha

**Affiliations:** Department of Cancer Chemoprevention, Chittaranjan National Cancer Institute (CNCI), Kolkata, West Bengal, India

**Keywords:** Orientin, SARS-CoV-2 spike, GRP78, *In silico* docking, MD Simulation, Phytochemical

## Abstract

The present wellbeing worry to the whole world is the outbreak of the severe acute respiratory syndrome coronavirus 2 (SARS-CoV-2), also called COVID-19. This global health crisis first appeared in Wuhan, China around December 2019 and due to its extremely contagious nature it had spread to almost 187 countries. Still now no effective method of treatment or vaccine is developed for controlling the disease. Therefore, the sole obliging strategy is to take precautionary measures by repurposing drugs from the pre-existing library of therapeutically potent molecules. In this situation of pandemic this repurposing technique may save the labour-intensive and tiresome process of new drug development. Orientin is a natural flavonoid with several beneficial effects. This phytochemical can be isolated from different plants like tulsi or holy basil, black bamboo, passion flowers etc. It's antiviral, anti-inflammation, vasodilatation, cardioprotective, radioprotective, neuroprotective, anticarcinogenic and antinociceptive effects are already established. In this research, it is intriguing to find out whether this molecule can interfere the interaction of SARS-CoV-2 spike glycoprotein and their host receptor GRP78. Our *in silico* docking and molecular dynamics simulation results indicate the binding of Orientin in the overlapping residues of GRP78 binding region of SARS-CoV-2 spike model and SARS-CoV-2 spike model binding region of GRP78 substrate-binding domain. Therefore, the results included in this research work provide a strong possibility of using Orientin as a promising precautionary or therapeutic measure for COVID-19.

## 1 Introduction

The latest emergence of pandemic threat of novel corona virus disease (COVID-19) by the pathogenic Severe Acute Respiratory Syndrome Coronavirus 2 is responsible for global risk of public health [1]. More than 13 million cases and around 285 thousand deaths have been recorded worldwide up to third week of July, 2020, which triggers the urgent need of active antiviral agent identification. The major symptoms of this disease include shortness of breath, fatigue, fever, muscle aches, dry cough and sometimes lead to pneumonia [1,2]. Patients with past medical history of other maladies like cancer, heart diseases, diabetes, asthma etc. along with elderly individuals and children below the age of 6 are in grave risk due to their compromised or weaker immune system. With the changing epicentres from Wuhan, China to countries like Italy, Spain and the USA this disease seems to increase its mortality rate [3, 4, 5]. A timely identification of causal agent called Severe Acute Respiratory Syndrome Coronavirus 2 (SARS-CoV-2) opened up new paths of antiviral research for COVID-19 treatment. SARS viruses have vivid plethora of host animals like birds, pangolins, domestic animals like dogs etc, whereas SARS-CoV-2 seems to be more pathogenic to human [6,7]. This is a virus with single stranded RNA genome of around 30 Kbs with low number of encoded proteins involved in structural and non-structural features of this member of genus *Betacoronavirus* [8,9,10]. The most prominent structural proteins are spike glycoprotein (S), envelope protein (E), membrane protein (M) and the nucleocapsid protein (N). On the other hand, proteases (nsp3 and nsp5) and RdRp (nsp 12) are the major non-structural proteins of this type of virus [8]. Out of these different types of protein spike protein is vital for viral attachment and entry to the host cells as they work as recognition factor. The earlier known SARS spike proteins have around 75%–81% sequence similarity with SARS-coronavirus 2 [11]. Though these RNA viruses have high mutational frequency, but very fewer differences in the spike proteins have been observed in emergent SARS-coronavirus 2 variants [12]. At the time of infection to the host cell the spike proteins are mostly in open state as the closed state of the same is less vulnerable to the antibodies [12]. The receptor binding domain of spike which faces outside part of virion shows distinctive "corona", or crown-like appearance [13]. The ectodomain of SARS-CoV-2 spike proteins comprise of two major domains: a N-terminal domain which is responsible for receptor binding and a C-terminal domain responsible for fusion with the host cells [14]. Binding affinity can be observed in some of these regions with host receptor proteins of SARS-CoV-2 like GRP78 [13].

The Glucose Regulating Protein 78 (GRP78) is a master chaperon protein which also known as Binding immunoglobulin protein (BiP) [15,16]. It generally acts when unfolded or misfolded proteins accumulate [17,18]. It has prominent role in cell death and differentiation as it causes the inactivation of enzymes involved in mentioned phenomena by binding to the lumen of the endoplasmic reticulum (ER) [19,20]. However, cell stress can also increase the chance of translocation of GRP78 from ER to cell membrane [20]. This makes GRP78 more susceptible to virus recognition and entry. The key viral recognition region of GRP78 is the substrate-binding domain (SBD) [19]. It is intriguing to find out molecular blocker of this substrate binding domain of GRP78 as well as the blocker for binding regions of spike proteins of SARS-CoV-2. Effective phytochemicals from natural resources may provide promising results as molecular blocker for GRP78 and spike proteins with no or less side effects. Orientin is a natural flavonoid can be found in many plants like Tulsi or Holy basil (*Ocimum sanctum*), Pheasant's eye (*Adonis vernalis*), Wilco (*Anadenanthera colubrine*), Cohoba (*Anadenanthera* peregrina*),* Black bamboo (*Phyllostachys nigra*), passion flowers (*Passiflora* species), Golden Queen (*Trollius* species), Bellyache Bush (*Jatropha gossypifolia*) etc [21]. Most of these plants are known for their medicinal values. Tulsi or Holy basil is a commonly used medicinal plant in ancient India. In ayurvedic literature it is mentioned as an effective remedy for many diseases like cough, common cold, malarial fever etc [22]. From the leaves of Holy basil Nair et al. and Uma Devi et al. successfully isolated orientin which has a molecular formula of C_21_H_20_O_11_ and a molecular weight of 448.3769 g/mol [21,23,24]. From different studies it is evident that orientin has antiviral, antioxidant, antiaging, anti-inflammation, vasodilatation and cardioprotective, radioprotective, neuroprotective, antiadipogenesis, anticarcinogeneic and antinociceptive effects which makes it promising therapeutic molecule [21]. Orientin is proved to possess antiviral activity against Para 3 virus which was demonstrated by Qiufeng et al. [25] and Li et al. [26]. Besides that, it also shows efficacy against Herpes Simplex Virus Type 2 (HSV-2) [27]. Therefore, it is fascinating to find out its antiviral effect against SARS-CoV-2.

In this study, we try to find out the *in silico* binding of orientin either with spike protein of SARS-CoV-2 or with GRP78. Here it is hypothesized that orientin has a scope to block the binding of spike protein and GRP78, thus it may restrict the recognition and entry of SARS-CoV-2 in GRP78 expressing host cells. Therefore, this report suggests that natural phytochemical orientin is a potential candidate for development of anti- SARS-CoV-2 drug.

## 2 Materials and method

### 2.1 Molecular modelling platform

Autodock Vina is used for protein-ligand docking and HADDOCK (ver. 4.2) is used for protein-protein interaction [28,29].

### 2.2 Preparation of protein

SARS-CoV-2 spike ectodomain structure (open state) is obtained from RCSB PDB (PDB id- 6VYB) and also a new spike protein model is generated from SARS-CoV-2 sequence which is downloaded from UniProt (UniProtKB-P59594) and modelled by Phyre2 web server [30]. Superposition and pairwise sequence alignment of SARS-CoV-2 spike ectodomain structure (open state) and NEW SARS-CoV-2 spike model is also done by Phyre2 webserver [30]. GRP78 SBD domain (PBD id. 5e85) is downloaded from RCSB PDB (Protein Data Bank).

### 2.3 Preparation of ligand

Orientin and various other plant derived compound like caffeic acid, isobavachalcone, lycorine, ellagic acid, galangin, an inhibitor of GRP78 i.e verrucosidin and clinically used anti-COVID19 drug chloroquine are screened against SARS-CoV-2 model and GRP78 SBD domain. 3D conformations of orientin, caffeic acid, isobavachalcone, lycorine, ellagic acid, galangin, verrucosidin and chloroquine are collected from PubChem database. Optimization of the 3d structure is done in Autodock tools (ver. 1.5.7) which utilizes Iterated Local Search global optimizer that based on the Broyden-Fletcher-Goldfarb-Shanno (BFGS) method [31,32]. Ligand preparation is done in Autodock tools under ligandprep function by removing water and adding hydrogen atom [33,34]. Energy minimalization is done by applying Gasteiger charges and AMBER force field with RMSD cut of 0.02 Å to obtain the most stable conformer [35].

### 2.4 *In silico* docking

*In silico* docking is performed to identify interacting residues responsible for ligand (orientin and other compounds) docking within the GRP78 SBD domain and as well as in the NEW SARS-CoV-2 spike model [36].

#### 2.4.1 Protein-protein docking

Docking between NEW SARS-CoV-2 spike model and GRP78 SBD domain is done by HADDOCK (ver. 4.2) webserver with easy interface and without any restrains [29,37]. In case of SARS COV-2 model spike protein N460, N487, D420, L455, K417, Y421, Y473, Y505, E484, N481, P479, E406, T478 are selected as active residues and for GRP78 SBD domain Q492, K447, S452, Q449, E427, I450, I450, S448, G430, T428, V429, S452, T458, V457, V490 are selected as active residues as mention in previous report [13,38]. Other than these residues all other surrounding amino acids are considered as passive residues. These active sides are directly involved in the interaction between two proteins where the passive residues are showing indirect interactions [37]. After docking the best docked clusters are ranked according to their HADDOCK score and all other parameters are represented with in separate statistical graphs.

The ranking of the clusters is based on the average score of the top 4 members of each cluster. The score is calculated as:

HADDOCK score = 1.0 × Evdw +0.2 × Eelec +1.0 × Edesol +0.1 × Eair

Where Evdw = intermolecular Van der Waals energy, Eelec = intermolecular electrostatic energy, Edesol = empirical desolvation energy, Eair = Restrain energy.

#### 2.4.2 Protein-ligand docking

Bindings of orientin, caffeic acid, isobavachalcone, lycorine, ellagic acid, galangin, verrucosidin and chloroquine with the new SARS-CoV-2 spike model and GRP78 SBD domain are executed by Autodock vina [28]. Each protein is prepared for docking by Autodock tools software where Gasteiger charges and hydrogen atoms is added and also make sure that water molecules are removed [39]. AMBER force field are applied for optimization of each structure [35]. For molecular docking grid boxes are also generated by the Autodock tools software, grid cubic boxes are generated using 0.492 Å spacing and with a dimension of 60 Å × 60 Å × 60 Å around each of the target protein.

Various docking model are generated and ranked according to their binding energy (Kcal/mol) and RMSD (upper bound) and RMSD (lower bound) values and represented in separate graph plots for orientin. The RMSD values are generated by this equation for two different structures a and b of same molecule-

$$\text{i})\;{\text{RMSD}}_{ab}=\max\left(\text{RMSD}\prime_{ab},\;\text{RMSD}\prime_{ba}\right)\text{,}$$

Where [40]

$$\text{ii)}\;RMSD{'}_{ab}=\sqrt{\frac{1}{N}\sum_{i}\min_{j}r_{ij}^{2}}\text{,}$$

Where N = no of heavy atom in structure a, min = over all atoms in structure *b* with the same element type as atom *i* in structure *a*.

Protein-Ligand Interaction Profiler (PLIP) web server of Technical University, Dresden is used to analyse the bond formation in docking complexes of NEW SARS-CoV-2 spike model-Orientin and GRP78 SBD domain-Orientin and two main interactions viz. hydrophobic interactions and hydrogen bonds are established [41]. Bond analyzation for Protein-protein docking complexes is performed in PIC (protein interaction calculator) and binding energy is predicted by PRODIGY for best model complexes of NEW SARS-CoV-2 spike model with GRP78 SBD domain and the residues in the interactions are tabulated [42,43,44].

### 2.5 Molecular dynamics simulation

The stabilities of the docked complexes are probed by finite temperature classical molecular dynamics [34]. Calculations are done in the simple point charge (SPC) water model using OPLS_2005 all atom force field (Optimized potentials for liquid simulation) as implemented in Desmond [45,46]. The ensemble type of the simulation is NPT with constant number of molecules (N), a defined temperature T (298K) and pressure P (1 bar). Nose-Hoover thermostat and Martyna-Tobias-Klein barostat are used to maintain the temperature and pressure of the system, respectively [45]. Protein ligand complexes are placed in a simulation box maintaining a minimum distance of 10 Å from the periodic boundary so that the complexes do not interact with their periodic image. The systems are minimized using the first four steps of the default five step minimization protocol as implemented in Schrodinger Desmond routine. Solute heavy atoms are restrained during the minimization. Production MD simulations are run for 40 ns. Binding free energies, root mean square deviations (RMSD) and root mean square fluctuations (RMSF) are calculated from the simulation trajectories. Residue numbering for GRP78 SBD and SARS-CoV-2 spike receptor binding domain are given as per the PDB structures 5e85 and 6vyb, respectively. Along with orientin and Verrucosidin 3D structure of chloroquine is also collected from PubChem database for MD simulation study.

## 3 Results and discussion

### 3.1 New SARS-CoV-2 spike model generation and sequence alignment with SARS-CoV-2 spike ectodomain structure

**Figure 1:**
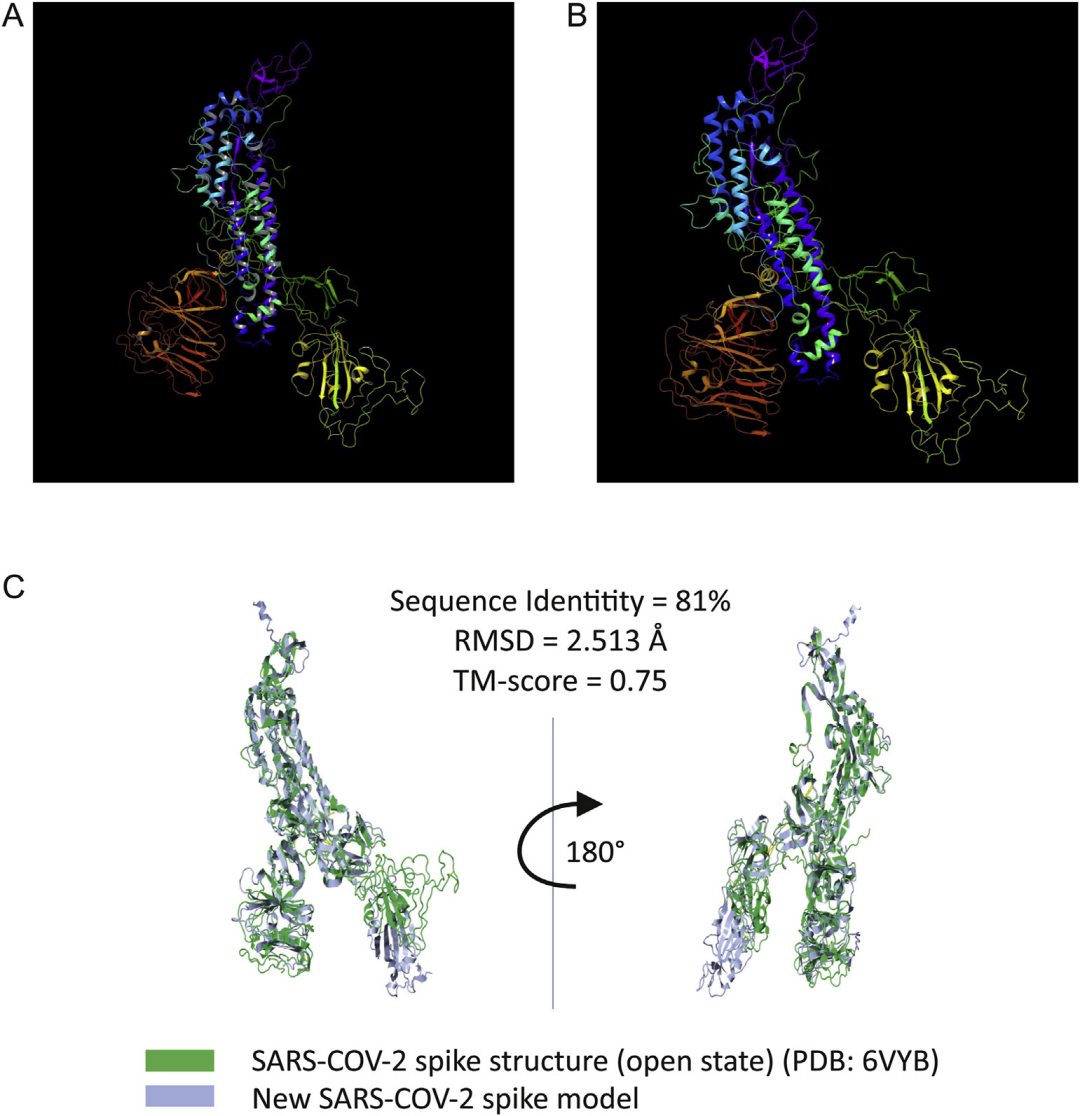
New SARS-CoV-2 spike model generated based on SARS-CoV-2 spike ectodomain structure (open state) (PDB: 6VYB). (A) Figure represents the 3D structure of SARS-CoV-2 spike ectodomain structure in its open state (colored cartoon). (B) Colored cartoon represents the structure of the new spike protein model of SARS-CoV-2 which is generated by Phyre2 web server. (C) Superposition of SARS-CoV-2 spike structure (green cartoon) and SARS-CoV-2 spike model (cyan cartoon) shows structural similarity.

**Figure 2:**
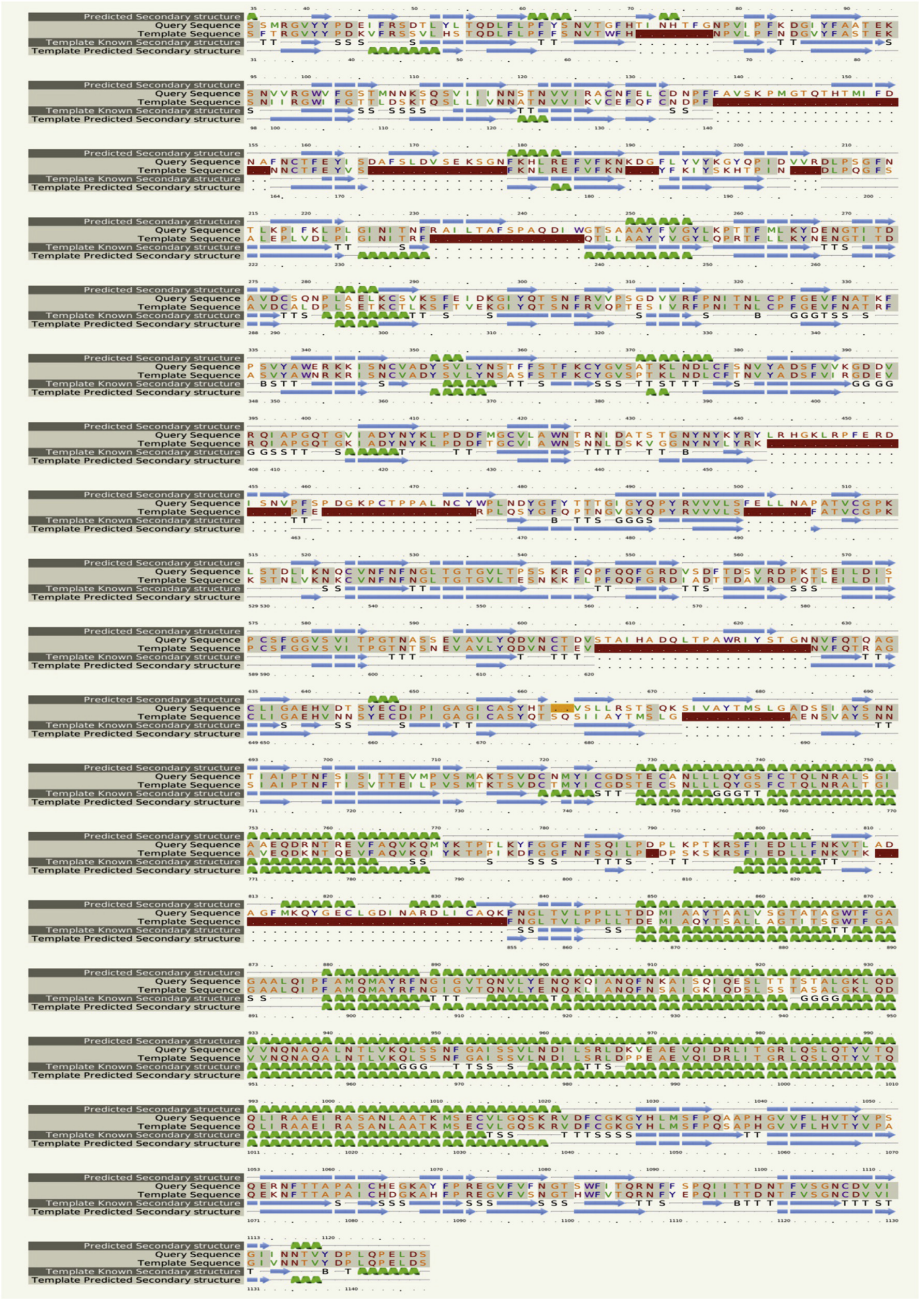
Pairwise representation of sequence alignment for the spike protein of SARS-CoV-2 (PDB: 6VYB) with new SARS-CoV-2 spike model. Template Sequence = sequence of SARS-CoV-2 spike (PDB: 6VYB), Query Sequence = New SARS-CoV-2 spike model. Identical residues in the alignment are highlighted with a grey background. Moderate conservations are indicated by thin grey bars whereas high degrees of conservations are indicated by large blocks. Red colored portion indicates insertion in query sequence relative to template and yellow colored portions indicates deletion in query sequence relative to template.

A potential 3D model of SARS-CoV-2 spike protein based on alignment to known protein structures is generated by Phyre2 (Protein Homology/analogY Recognition Engine V 2.0). This method can generate accurate protein models of about 70% of the domain similarity with a known structure where the core of the protein shows 2-4Å root mean square deviation from the native. Remote homology detection techniques such as profile matching and hidden Markov model (HMM) matching is used to detect and align protein sequences. This efficient technique is able to generate reliable models of proteins even if they have considerable divergence over evolutionary time. The newly generated SARS-CoV-2 spike model sequence is close to the SARS-CoV-2 spike ectodomain structure (open state) (PDB: 6VYB) with 81% sequence identity. Figure 1A shows colored cartoon of the known structure of SARS-CoV-2 spike ectodomain (PDB: 6VYB) which serves the roll of template for the new model generation. Figure 1B represents newly generated 3D model of SARS-CoV-2 spike (colored cartoon). The Root Mean Square Deviation (RMSD) between the two structures is 2.513 Å. The template modelling score or TM-score is 0.75 which indicates significant similarity between the structures of SARS-CoV-2 spike ectodomain and newly generated SARS-CoV-2 spike model. Figure 1C shows the superposition of SARS-CoV-2 spike model (blue cartoon) and SARS spike structure (PDB ID: 6VYB) (green cartoon). Two views are shown with a vertical axis rotation of 180°. In Figure 2, pairwise representation of sequence alignment for the spike protein of SARS-CoV-2 (PDB: 6VYB) with new SARS-CoV-2 spike model generated by Phyre2 is based on HMM-HMM matching. Here, the template sequence is represented by the sequence of SARS-CoV-2 spike (PDB: 6VYB), whereas for query sequence new SARS-CoV-2 spike model sequence is used. In this illustration, along with the simple pairwise representation of the alignment in FASTA format extra rows are present which entitled "Template known secondary structure", "Template predicted secondary structure" and "Predicted secondary structure". For generating the alignment, both the predicted secondary structure of new SARS-CoV-2 spike model sequence and the known/predicted secondary structure of the SARS-CoV-2 spike (PDB: 6VYB) template are used in conjunction with the sequence information. In this alignment, characters like G,I,T,B,S of "Template known secondary structure" represent 3-turn helix (3_10_ helix), 5-turn helix (π helix), hydrogen bonded turn, residue in isolated β-bridge and bend respectively. Grey highlights are the representation of identical residues in the alignment where thin grey bars indicate moderate conservation and large grey blocks indicate high degree of conservation of the residues in new SARS-CoV-2 spike model sequence. On the other hand, no highlights are the indication of nonconserved sequences. Secondary structure can also be predicted from the representation of alignment as the green helices are signifying α-helices, blue arrows are representing β-strands and faint lines are indicating coil of the template structure of SARS-CoV-2 spike and predicted structure of new SARS-CoV-2 spike model. Insertion and deletion in new SARS-CoV-2 spike model sequence relative to template sequence of SARS-CoV-2 spike structure is represented by red and yellow highlighted region respectively. Residues like 71–77, 138–156, 166–178, 190–192, 205–207, 232–245, 443–458, 462–475, 551–556, 607–626, 673–682, 790, 811–836 are inserted in new SARS-CoV-2 spike model sequence (query sequence) by Phyre2 based prediction, whereas 677 and 678 residues of template SARS-CoV-2 spike (PDB: 6VYB) sequence are deleted in the in new SARS-CoV-2 spike model sequence. Therefore, a complete ectodomain model (open state) of SARS-CoV-2 is generated to fill the lacuna of insufficient sequence data of known SARS-CoV-2 spike ectodomain structure (PDB: 6VYB) obtained from protein data bank. This newly generated model is further used for other *in silico* experiments mentioned in this research work.

### 3.2 Identification of residues for binding of substrate-binding domain (SBD) of GRP78 with new SARS-CoV-2 spike model

**Figure 3:**
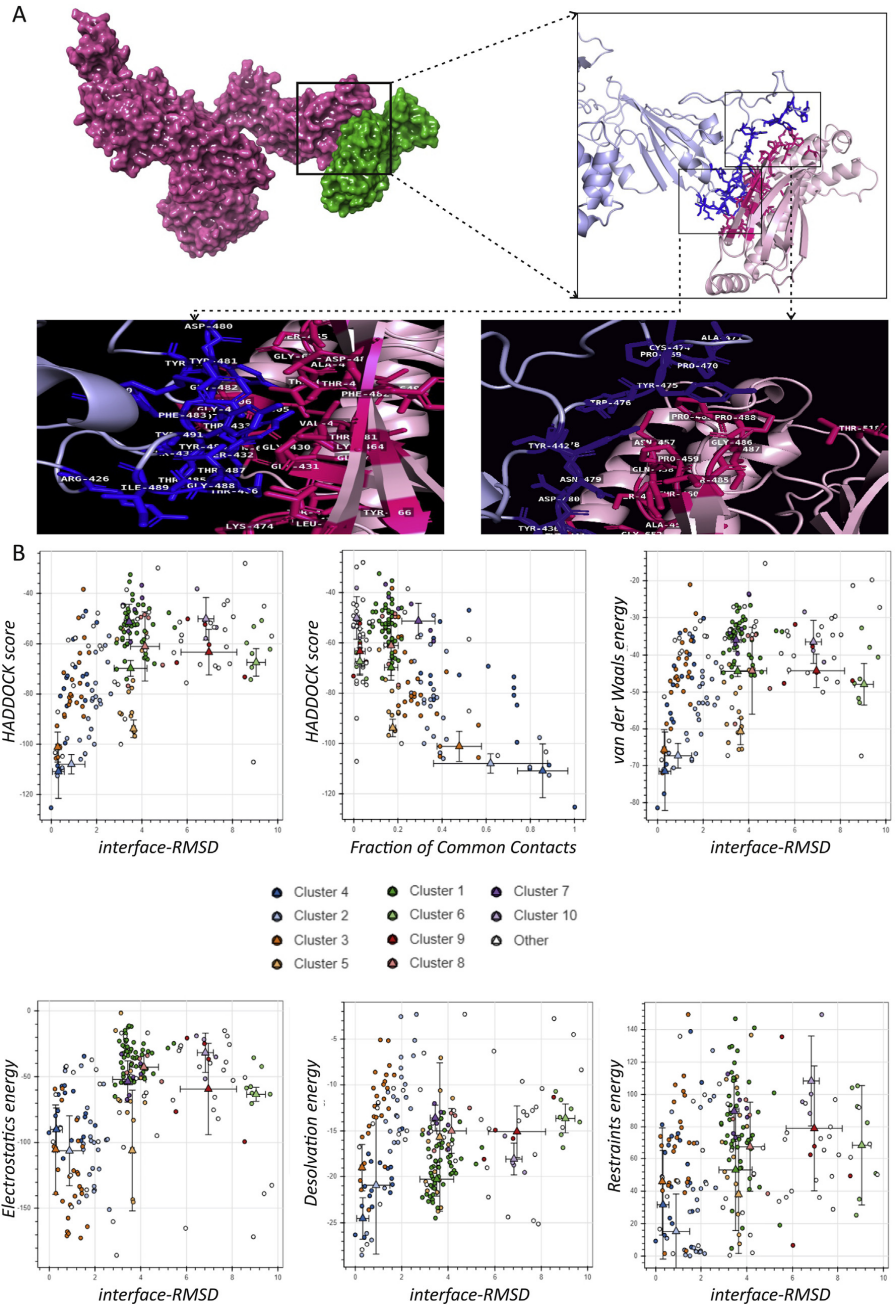
Dockking of new SARS-CoV-2 spike model with substrate-binding domain (SBD) of GRP78. (A) Figure shows the proposed binding mode of the host cell GRP78 SBD domain (green surface) and the New SARS-CoV-2 spike model (violet surface). Enlarged panel represents the interaction of amino acids from the GRP78 SBD (pink cartoon) and the New SARS-CoV-2 spike model (blue cartoon). Interactive amino acids are labelled and interactions are represented in blue sticks (for new SARS-CoV-2 spike model) and red sticks (for GRP78 SBD domain) in the enlarged panel. (B) Figure represents graphics based on water-refined models generated by HADDOCK. The clusters demarcated by different colors in graph and various structural analysis by interface-RMSD (i-RMSD), Fraction of Common Contacts (FCC), Van der Waals energy, Electrostatics energy, Desolvation energy and Restraints energy are made with respect to the best HADDOCK model with lowest HADDOCK score.

HADDOCK software is used to perform GRP78- SARS-CoV-2 spike model docking. Figure 3A shows the best-formed complex from the docking experiment with green surface representing substrate binding domain of GRP78 and violet surface representing the new SARS-CoV-2 spike model. Enlarged figures represent cartoons of docking where stick models are marked with the interacting residues for docking. HADDOCK clustered 157 structures in 10 clusters, which represent 78 % of the HADDOCK generated water-refined models. Rest of that percentage is considered as “other”. The maximum number of models considered for clustering is 200. According to HADDOCK out of the top 10 clusters “cluster 4” is the most reliable as it is showing best binding of GRP78 SBD with SARS-CoV-2 spike model which has low docking score (HADDOCK score) of -110.8 +/- 9.2 and best RMSD from the overall low-energy structures of different clusters. The RMSD value of the lowest-energy structure is 0.5 +/- 0.3. From the docking trials it is proved that there is possibility of fitting the GRP78 SBD to the spike model as it shows binding affinity (predicted by PRODIGY) of -12.9 kcal/mol Table 1 and Table 2 represent the interactions of spike model with the substrate-binding domain of GRP78 by twenty six hydrogen bonds (through GLU 427, SER 432, THR 433, TYR 466, TRP 476, ASN 479, TYR 484, THR 486, GLY 488, TYR 491 and GLN 609) and eight hydrophobic interactions (through TYR 436, TYR 442, PRO 470, TYR 475, TRP 476 and TYR 484). The average hydrogen bond length for the docking trial of the spike model is 3.14 ± 0.33 Å. In Figure 3B, graphs represent the clusters indicated in color. These clusters are calculated based on the interface-ligand RMSDs calculated by HADDOCK, where the interfaces are defined based on all observed contacts. The various structural analyses are made with respect to the best HADDOCK model. Therefore, for the best-docked complex values of Van der Waals energy is -71.5 +/- 9.2, electrostatic energy is -90.0 +/- 14.2, desolvation energy is -24.5 +/- 2.0, restraints violation energy is 31.7 +/- 29.0. In the graphs of Figure 3B, best cluster of docked models is defined by lowest HADDOCK score in respect to lowest interface RMSD (i-RMSD) value, lowest HADDOCK score in respect to highest value of fraction of common contacts, lowest Van der Waals score in respect to lowest interface RMSD, highest electrostatics energy in respect to lowest interface RMSD, lowest desolvation energy in respect to lowest interface RMSD and lowest restraints energy in respect to lowest interface RMSD. Therefore, cluster 4 is considered as best cluster as per the mentioned parameters and best binding model of cluster 4 is represented in Figure 3A. Hence, it is evident from the best docked model that predicted binding residues of SARS-CoV-2 spike model lies between the sequences of 427–609 amino acids, whereas 427–652 amino acid sequence is the region for the binding of GRP78 SBD.

**Table 1:** The interactions formed between GRP78 SBD domain and the new SARS-CoV-2 spike model based on docking with HADDOCK. Table represents the hydrogen bonds generated between the new SARS-CoV-2 spike model and host cell-surface GRP78 SBD during HADDOCK based docking.

HYDROGEN BOND = 26		
Residue from New SARS-COV2 Spike	Residue from GRP-78	Bond Length(Å)
SER 432	GLY 430	3.17
THR 433	GLU 606	3.13
ASN 479	GLY 652	2.29
ASN 479	GLY 652	2.29
ASN 479	GLY 652	3.39
ASN 479	GLY 652	3.39
TYR 484	THR 428	3.37
GLY 488	TYR 466	3.13
GLU 427	THR 486	3.21
GLU 427	THR 486	3.21
TYR 466	THR 486	3.04
GLN 609	SER 432	3.43
GLN 609	SER 432	3.43
TRP 476	ASN 457	3 .25
ASN 479	THR 460	3.12
ASN 479	THR 460	3.12
ASN 479	THR 460	3.45
ASN 479	THR460	3.45
THR 486	GLU 427	2.59
THR 486	THR473	3.35
TYR 491	ASP 483	3.06
ASN 479	THR 460	3.12
ASN 479	THR 460	3.45
THR 486	THR 473	3.35
TYR 491	THR 481	2.89
TYR 484	THR 651	2.83

**Table 2:** The interactions formed between GRP78 SBD domain and the new SARS-CoV-2 spike model based on docking with HADDOCK. In Table hydrophobic interactions between residues of new SARS-CoV-2 spike model and host cell-surface GRP78 SBD are represented.

Hydrophobic interaction = 8	
Residue from New SARS-COV2 Spike	Residue from GRP-78
TYR 436	VAL 429
TYR 436	ALA 454
TYR 442	PRO 459
PRO 470	PRO 489
TYR 475	PRO 488
TYR 475	PRO 489
TRP 476	PRO 489
TYR 484	VAL 429

### 3.3 Binding of orientin in GRP78 binding region of SARS-CoV-2 spike model and SARS-CoV-2 spike model binding region of GRP78

**Figure 4:**
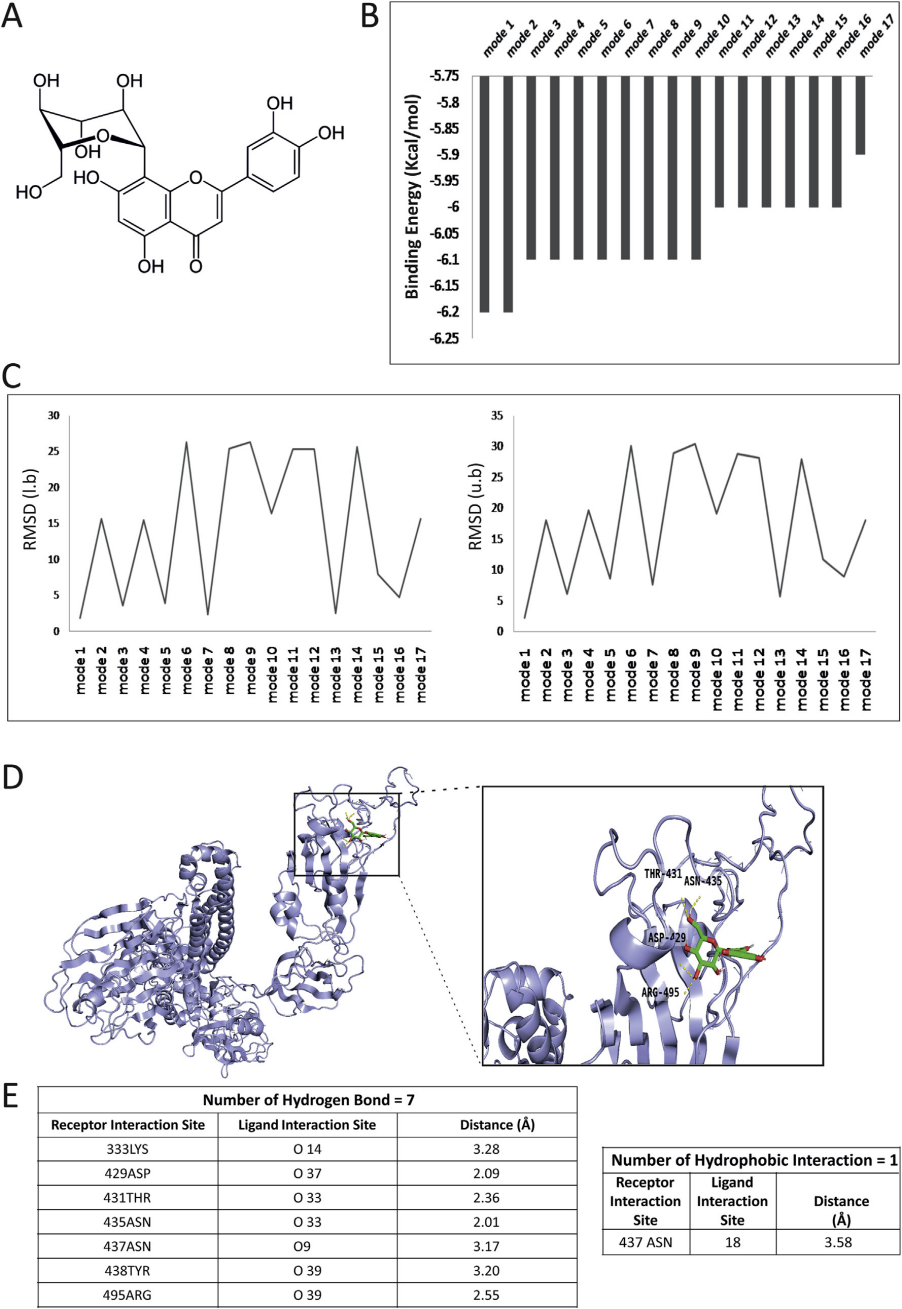
Docking of Orientin with New SARS-CoV-2 spike model. (A) Figure shows chemical structure of Orientin. (B) Graph represents modes of binding of Orientin with New SARS-CoV-2 spike model where lowest binding energy represents best mode of binding. (C) Figure shows graphical representation of Root Mean Square Deviation (RMSD) lower bound (l.b) and upper bound (u.b) values for different modes of binding as per the results of docking of Orientin with New SARS-CoV-2 spike model in the platform of Autodock vina. (D) The illustration represents binding of Orientin (green sticks) with SARS-CoV-2 spike model (blue cartoon), whereas in the enlarged panel interactive amino acids of SARS-CoV-2 spike model are represented. (E) Interaction sites for receptor (SARS-CoV-2 spike model) and ligand (Orientin) for hydrogen bond formations and hydrophobic interaction are tabulated. Bond distances are mentioned in the table.

**Figure 5:**
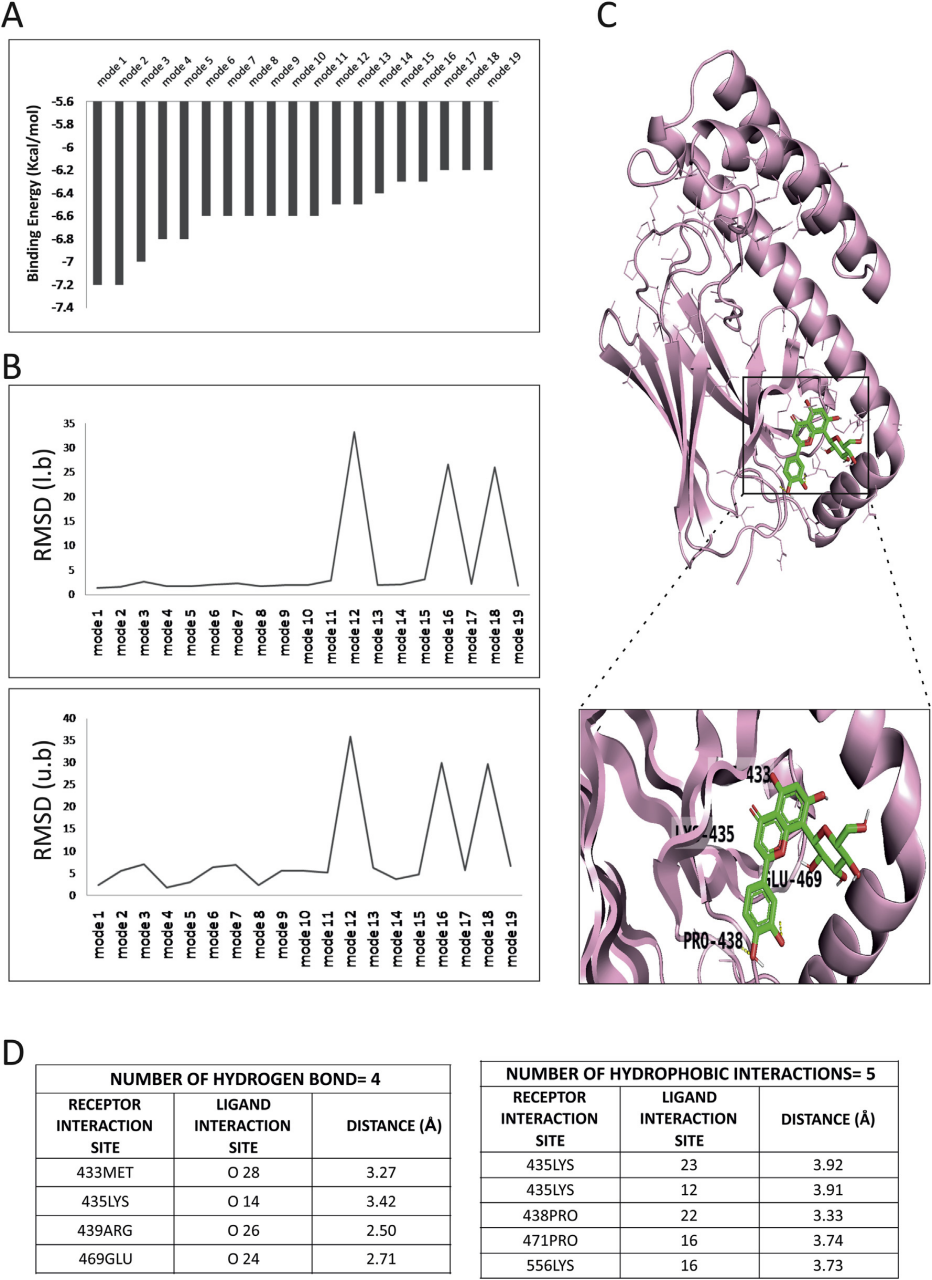
Dockking of Orientin model with substrate-binding domain (SBD) of GRP78. (A) Graph represents binding modes of Orientin with SBD of GRP78 where best mode of binding is determined by lowest binding energy. (B) Figure shows graphical representation of RMSD l.b and RMSD u.b values for different modes of binding as per the results of docking of Orientin with SBD of GRP78. (C) The figure illustrates binding of Orientin (green sticks) with SBD of GRP78 (pink cartoon), whereas in the enlarged panel interactive amino acids of GRP78 are represented. (D) Figure shows tabulation of interaction sites for receptor (SBD of GRP78) and ligand (Orientin) for hydrogen bond formations and hydrophobic interaction. Bond distances are mentioned in the table.

The water-soluble flavonoid orientin is a *C*-glycoside which has the IUPAC name of 2-(3,4-dihydroxyphenyl)-5,7-dihydroxy-8-[(2S,3R,4R,5S,6R)-3,4,5-trihydroxy-6-(hydroxymethyl)oxan-2-yl]chromen-4-one [21]. Figure 4A represents the chemical structure of orientin which consists of mostly phenol groups with two ether groups and one ketone group. After determination of residues responsible for interaction between SARS-CoV-2 spike model and GRP78, it is intriguing to find out whether orientin can bind within the domain of SARS-CoV-2 spike model where GRP78 SBD can also bind. On the other hand, binding affinity of orientin with SBD or substrate binding domain of GRP78 is also assessed by molecular docking. As this substrate binding domain is essential for SARS-CoV-2 spike model binding with GRP78, binding of orientin in the same region may infer blockage in SARS-CoV-2 spike and GRP78 SBD interaction. After building the protein structures by the help of phyre2 webserver the structures are completed for docking using AutoDock tools by adding polar hydrogen and Gasteiger charges for global optimization and again processed for energy minimization. After completion of docking with 3D model of orientin all the docking poses are categorized according to their affinity, RMSD values. Among all these conformations the best pose is considered as the binding model for orientin and SARS-CoV-2 spike model protein, as well as for orientin and GRP78 SBD. The best binding model has the highest binding affinity as well as low RMSD values, as the higher binding affinity indicates low chance of dissociation as well as high chance of binding probability between protein and ligand. Besides this, RMSD indicates the measurement of the difference between a ligand's crystal conformation and docking prediction, lower RMSD specifies less average distance between the native atom position and the position of that same atom after docking [47]. This RMSD values are divided into two sub groups named as RMSD upper bound (u.b) and RMSD lower bound (l.b). RMSD u.b matches each atom in one conformation with itself in the other conformation, ignoring any symmetry, whereas RMSD l.b matches each atom in one conformation with the closest atom of the same element type in the other conformation. In case of docking between orientin and new SARS-CoV-2 model spike, Autodock vina software is used. After docking 17 best docking poses are considered for comparison. All different binding modes are represented according to their binding affinity in Figure 4B and also represented in separate RMSD graphs for both RMSD u.b and RMSD l.b groups (Figure 4C) and among these mode-1 (Figure 4D) is the best docked model because of having high affinity score (-6.2 kcal/mol) and lower RMSD l.b value 1.813 and RMSD u.b value of 2.215 than any other modes. The best binding mode of orientin and SARS-CoV-2 spike model represents it's binding within the region ranging from residue no. 333–495 (Figure 4D). The interacting residues between Orientin and SARS-CoV-2 spike model (Figure 4E) shows seven hydrogen bonds (through LYS 333, ASP 429, THR 431, ASN 435, ASN 437, TYR 438, ARG 495) with an average hydrogen bond distance of 2.66 ± 0.61 Å and one hydrophobic interaction (ASN 437). This indicates the binding of orientin in the same region of SARS-CoV-2 spike model where GRP78 can bind, as the interacting residues of SARS-CoV-2 spike model – GRP78 docking ranges from 427-609 amino acids of SARS-CoV-2 spike model (Table 1) which overlaps with the orientin binding region of SARS-CoV-2 spike model. On the other hand, Autodock vina is also used for binding prediction between GRP78 SBD and orientin. Models with different binding affinity and different RMSD are represented in separate graphs (Figures 5A and 5B). Top 19 binding modes are represented and among these mode-1 is the best binding model having highest binding affinity of -7.2 Kcal/mol and lowest RMSD l.b value of 1.409 as well as lowest RMSD u.b value of 2.393. the interacting residues of orientin within GRP78 SBD are ranging between 433-469 amino acids (Figure 5C) and have four hydrogen bonds (through MET 433, LYS 435, ARG 439, GLU 469) with average distance of 2.95 ± 0.29 Å and five hydrophobic interactions (through LYS 435, PRO 438, PRO 471, LYS 556) with average distance of 3.72 ± 0.20Å (Figure 5D). In contrast, binding region of GRP78 to SARS-CoV-2 spike model lies between 427-652 amino acid region of SBD (Table 1). Overlapping binding region of GRP78 SBD docking with SARS-CoV-2 spike model and orientin indicates the interference in SARS-CoV-2 spike binding to GRP78 SBD in presence of orientin.

Docking with orientin and other known phytochemicals against COVID-19 i.e caffeic acid, isobavachalcone, lycorine, ellagic acid, galangin and inhibitor of GRP78 verrucosidin suggest that orientin is comparable or better than the known anti- SARS-COV-2 phytochemicals in terms of *in silico* binding with GRP78 SBD and SARS-COV-2 spike model (Table 3) [48,49,50,51,52,53,54,55,56,57,58,59,60,61,62,63]. The *in silico* docking results suggest the binding of orientin with SARS-COV-2 spike model's receptor binding domain and also with GRP78 SBD with better affinity than the mentioned phytochemicals. Other than this, compared to the GRP78 inhibitor verrucosidin higher binding affinity of orientin is observed in case of docking with both GRP78 SBD and SARS-COV-2 spike model. Binding affinity of SARS-COV-2 spike model with caffeic acid, isobavachalcone, lycorine, ellagic acid, galangin and verrucosidin are -5.1 Kcal/mol, -6.0 Kcal/mol, -5.7 Kcal/mol, -6.0 Kcal/mol, -5.8 Kcal/mol, -6.1 Kcal/mol respectively, lower than the binding affinity of orientin with SARS-COV-2 spike model (-6.2 Kcal/mol) (Table 3). On the other hand, binding affinity of caffeic acid, isobavachalcone, lycorine, ellagic acid, galangin and verrucosidin with GRP78 SBD domain are -5.0 Kcal/mol, 6.6 Kcal/mol, 7.2 Kcal/mol, -6.4 Kcal/mol, -6.1 Kcal/mol, -5.9 Kcal/mol respectively, comparable or lower than the binding affinity of orientin with GRP78 SBD domain (-7.2 Kcal/mol) (Table 3). Binding results also suggest that orientin can bind more efficiently with GRP78 than its natural inhibitor verrucosidin which in turn advocate the potential of orientin as an efficient binding molecule of GRP78 SBD. Other than these, orientin also shows better binding affinity than the repurposed anti-COVID-19 drug chloroquine as the docking results of it with GRP78 SBD and SARS-COV-2 spike model confirms the binding energy -5.1 Kcal/mol and -5.4 Kcal/mol respectively (Table 3) [64].

**Table 3:** Table represents binding affinity and best docked conformations of different phytochemicals, GRP78 inhibitor verrucosidin and clinically used anti-COVID19 drug chloroquine with SARS-CoV-2 spike model and GRP78 surface binding domain.

Compound name	Reference	Natural Source	Compound Structure	Binding Affinity (Kcal/mol)		Best docked conformation	
				with COVID-19	with GRP-78	with SARS-CoV-2 spike	with GRP78
Orientin	[48,49]	Tulsi or Holy Basil (*Ocimum sanctum),*Pheasant's eye (*Adonis vernalis)*, wilco (*Anadenanthera colubrina)*, bamboo leaves (*Phyllostachys nigra)*, passion flower (*Passiflora actinia*) etc.	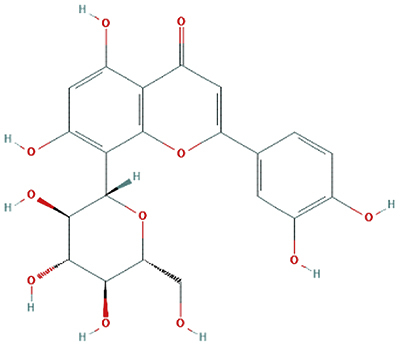	-6.2	-7.2	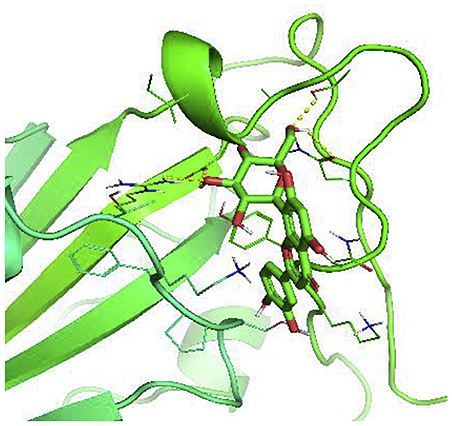	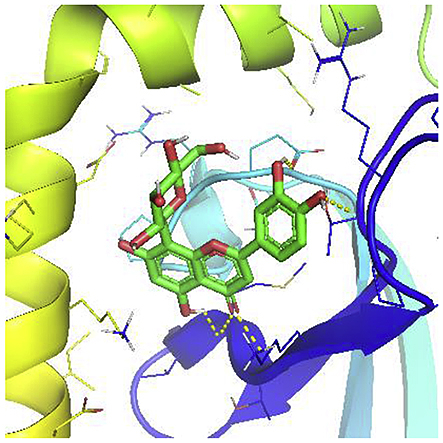
caffeic acid	[50,51,52]	Bark of Eucalyptus (*Eucalyptus globulus*), Coffee (*Coffea arabica*), Sunflower seeds (*Helianthus annuus*)	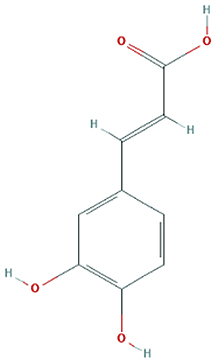	-5.1	-5.0	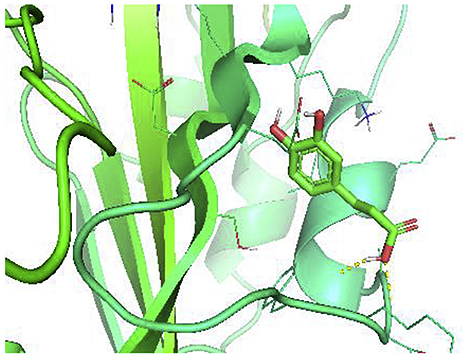	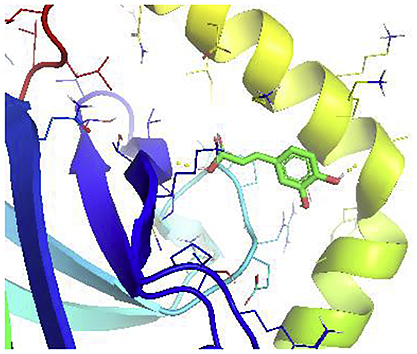
Isobavachalcone	[53,54]	Plants of Moraceae and Fabaceae family	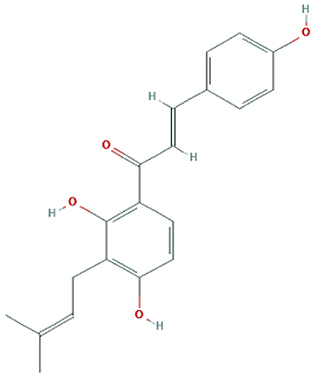	-6.0	-6.6	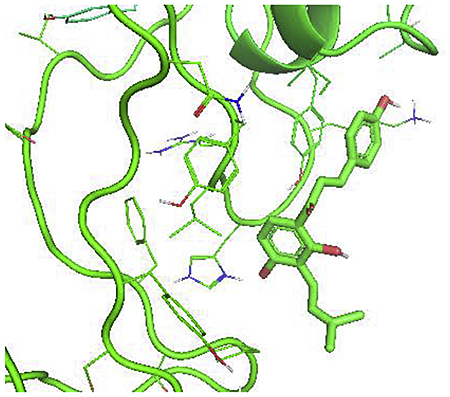	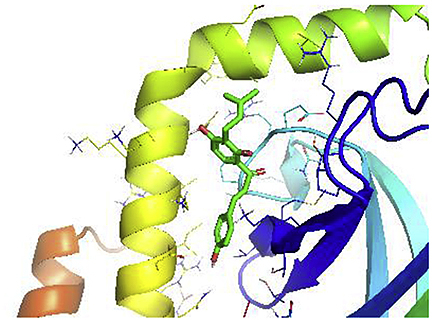
Lycorine	[52,54,55]	Bush lily (*Clivia miniata*), Surprise lilies (*Lycoris squamigera*), and Daffodils (*Trumpet narcissus*)	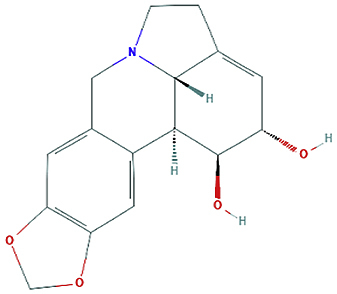	-5.7	-7.2	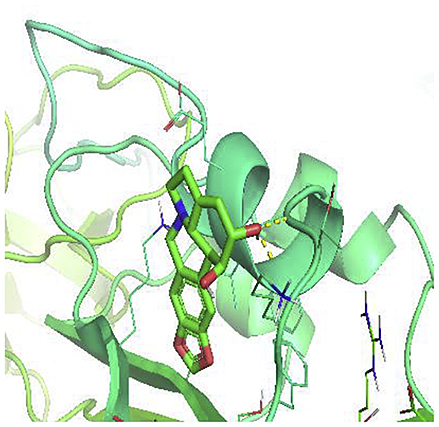	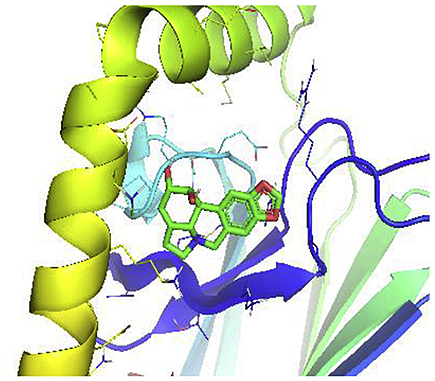
Ellagic acid	[56,57,58,59,60]	Raspberries (*Rubus idaeus*), Strawberries (*Fragaria ananassa*), Cherries (*Prunus avium*), Blackberries (*Rubus fruticosa*), and Walnuts (*Juglans regia*)	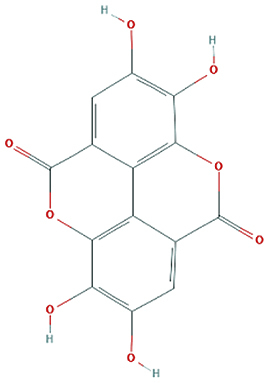	-6.0	-6.4	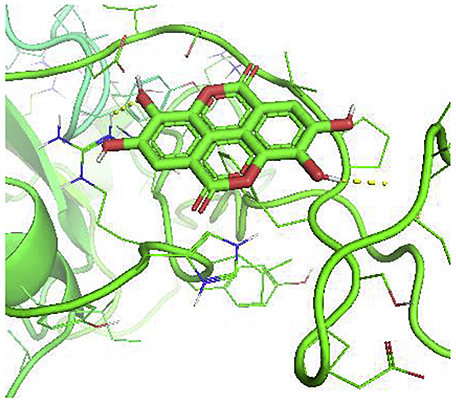	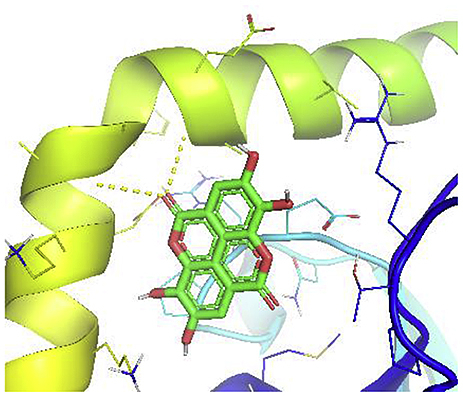
Galangin	[60,61]	Lasser galangal (*Alpinia officinarum*), Honey	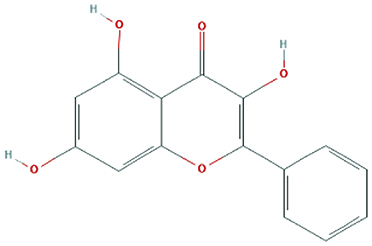	-5.8	-6.1	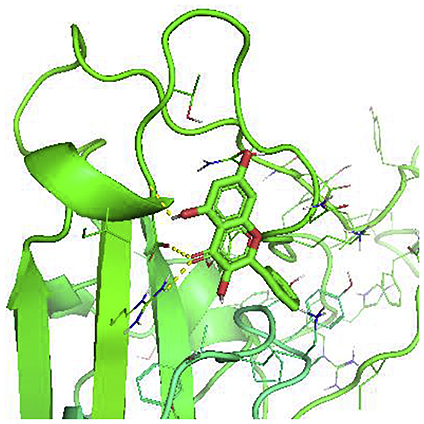	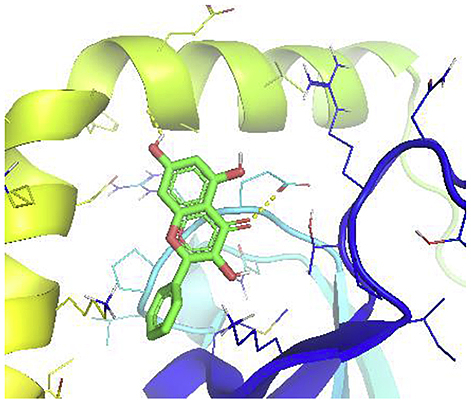
Verrucosidin	[62,63]	*Penicillium aurantiogriseum*	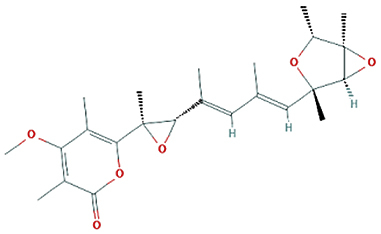	-6.1	-5.9	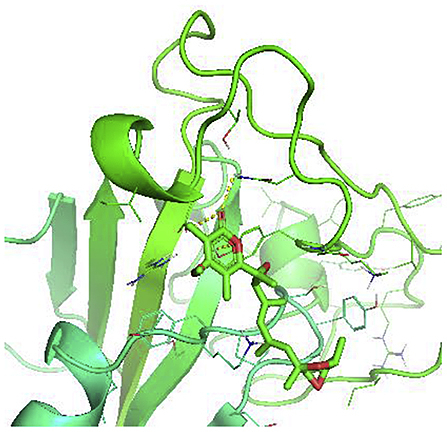	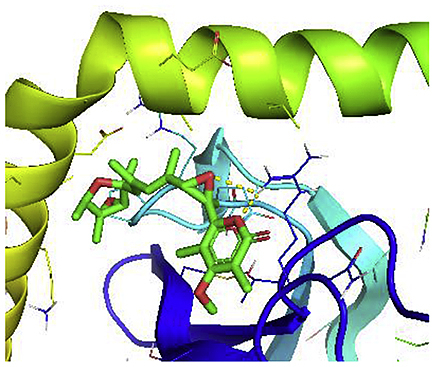
Chloroquine	[64]	Semi-synthetic derivative of Quinine isolated from *Cinchona officinalis*	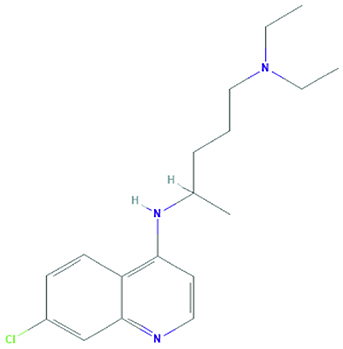	-5.4	-5.1	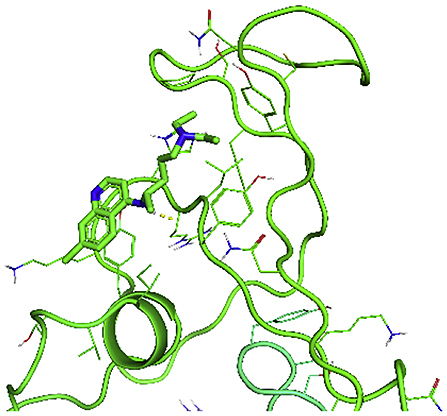	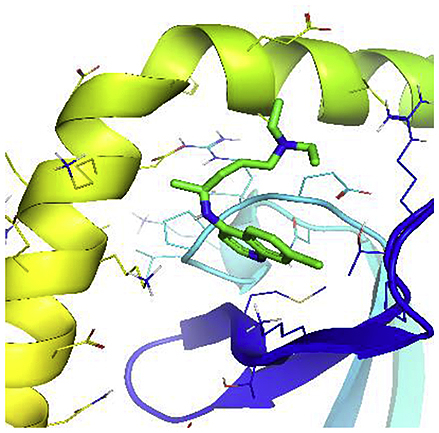

**Figure 6:**
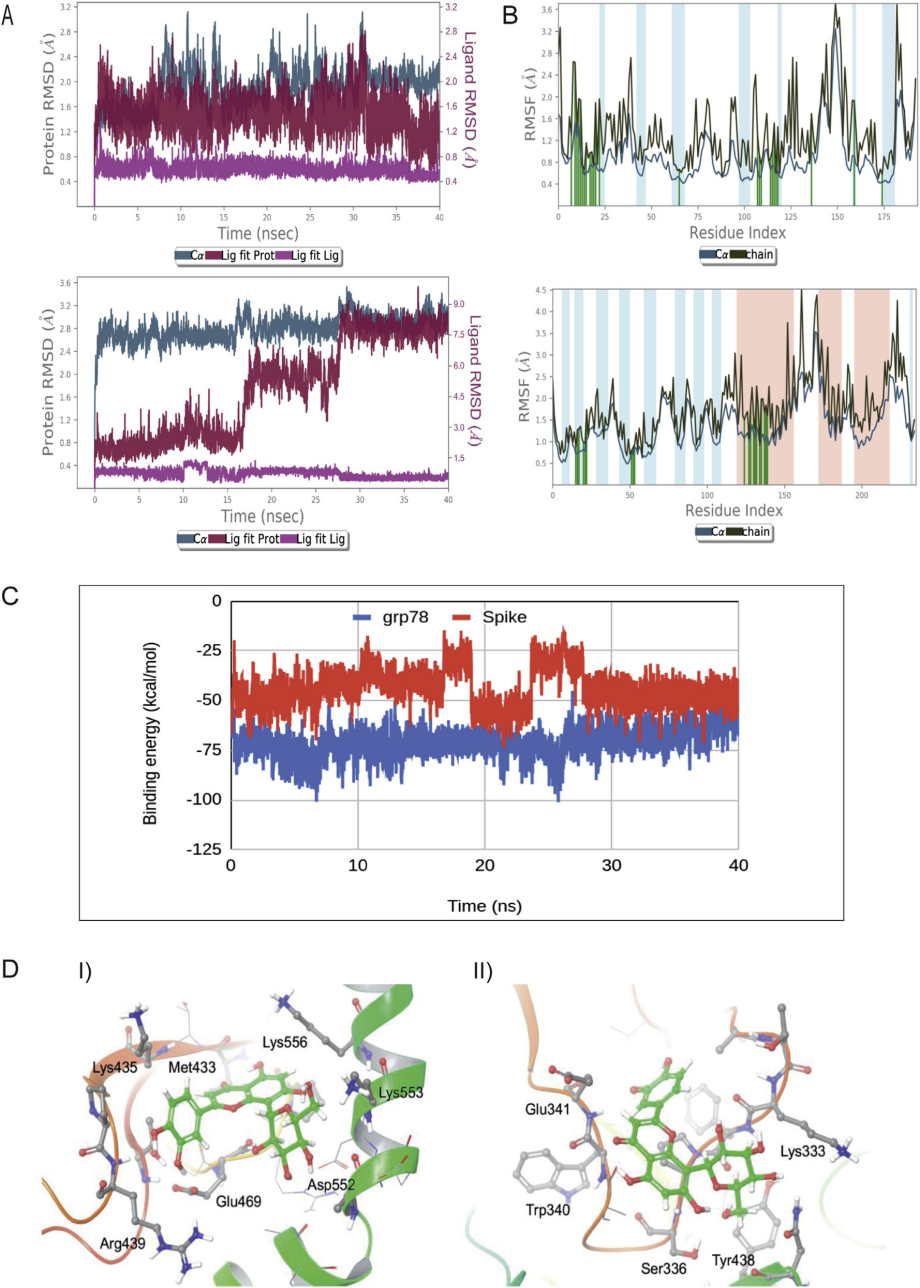
MD Simulation of Orientin with GRP78 SBD and SARS-CoV-2 spike model. (A) Graph represents overall structural changes (RMSD) in the orientin bound GRP78 (top) and spike protein (bottom) complexes over 40 ns time. (B) Graph shows root mean square fluctuations (RMSF) in the backbone and side chains of SARS-CoV-2 spike receptor binding domain (top) and GRP78 SBD. Ligand contacts are shown in green lines. (C) Graph shows binding free energies for orientin binding with the GRP78 SBD (blue) and SARS-CoV-2 spike model (red) over 40 ns of simulation trajectories. (D) Figure shows snapshots of MD simulation after 40 ns. Orientin (green stick model) in the binding site of GRP78 (I) and SARS-CoV-2 spike model receptor binding domain (II). Amino acid side chains in the binding sites are shown.

From the MD simulation trajectories, orientin is found to remain bound with its targets, SBD (substrate binding domain) of GRP78 and receptor binding domain of SARS-CoV-2 spike, for the whole duration of 40 ns unrestrained simulation indicating stable interaction (Supporting information video 1–4). Overall structural deviations of the complexes are depicted in Figure 6A in terms of root mean square deviations (RMSD). It is evident from the figure that the proteins quickly reach their equilibrium geometries within a few picoseconds. Maximum structural changes observed for GRP78 SBD and the spike domain are ~2 Å and ~2.8 Å, respectively. Very low RMSD value of ligand with respect to ligand (Figure 6A) indicates that Geometry of orientin remains unaltered during the simulation. Ligand RMSD with respect to protein (Figure 6A) suggests that the orientation of orientin in the binding site of spike protein changed with time and after 27 ns reached a stable conformation. Whereas, for GRP78, the docked conformation of orientin remained in equilibrium with the protein for the whole duration of simulation. Residue-wise fluctuations (RMSF) in the backbone and the side chain of both the proteins are given in the Figure 6B.

Supplementary video related to this article can be found at https://doi.org/10.1016/j.heliyon.2021.e05923

The following is the supplementary data related to this article:

SARS-CoV-2 spike_Chloroquine_MD simulation SARS-CoV-2 spike_Chloroquine_MD simulation

SARS-CoV-2 spike_Verrucosidin_MD simulation SARS-CoV-2 spike_Verrucosidin_MD simulation

GRP78_Verrucosidin_MD simulation GRP78_Verrucosidin_MD simulation

SARS-CoV-2 spike_Orientin_MD simulation_01 SARS-CoV-2 spike_Orientin_MD simulation_01

Binding free energies of orientin with the SBD of GRP78 and receptor binding domain (RBD) of SARS-CoV-2 spike are computed from the simulation trajectories and is shown in Figure 6C. Average binding free energies for GRP78 SBD and SARS-CoV-2 spike model as obtained from the last 10 ns of the trajectories are -66.14 ± 5.96 kcal/mol and -48.02 ± 5.49 kcal/mol, respectively. Binding with the GRP78 is energetically more favourable than the spike protein.

**Figure 7:**
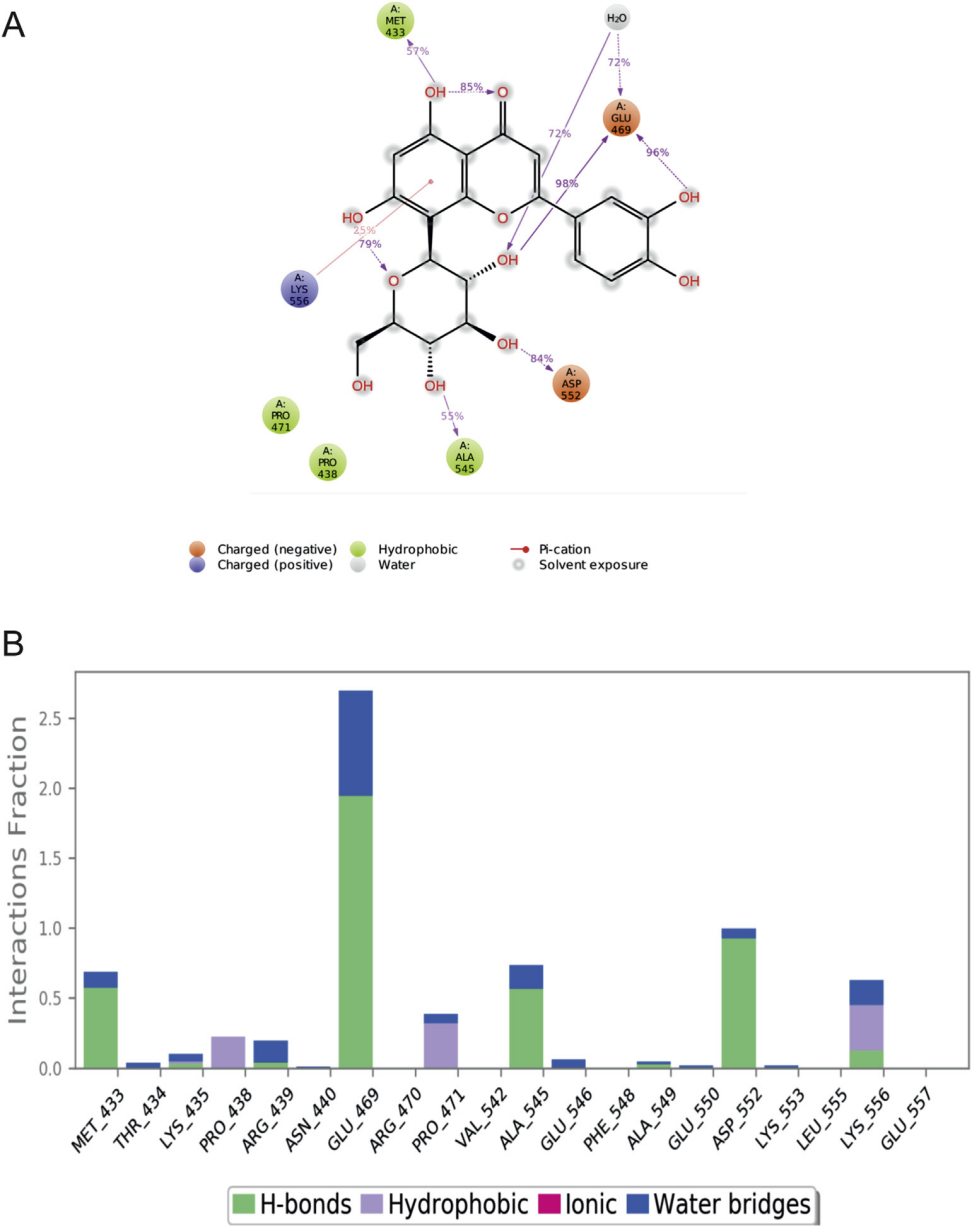
Different interactions of Orientin and GRP78. (A) Figure represents major interacting residues of GRP78 with orientin. (B) Bar graph shows interaction fractions of different types of interactions in orientin-GRP78 complex.

**Figure 8:**
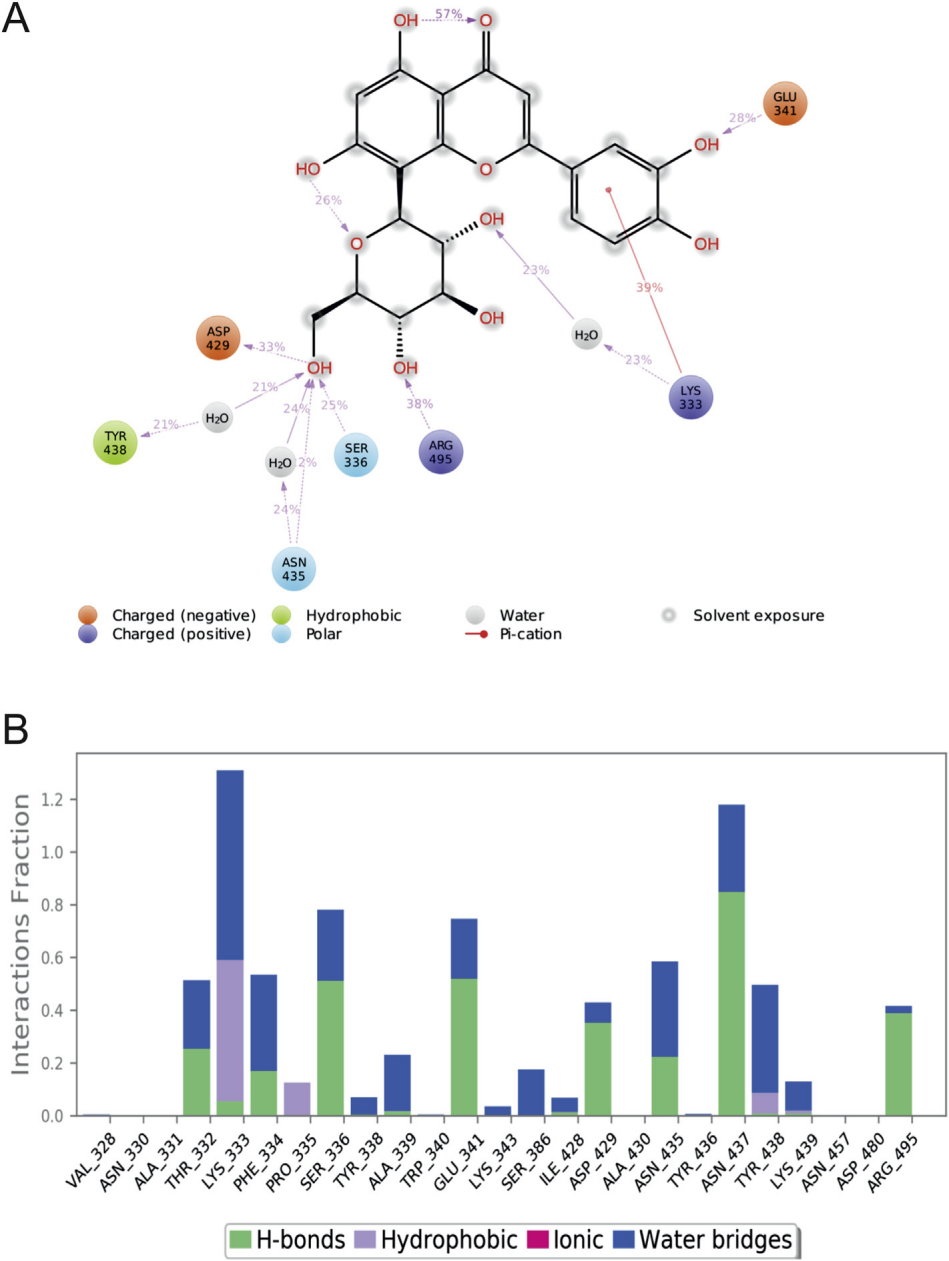
Different interactions of Orientin and SARS-CoV-2 spike. (A) Figure represents major interacting residues of spike protein with orientin. (B) Bar graph shows interaction fractions of different types of interactions in orientin-spike protein complex.

**Figure 9:**
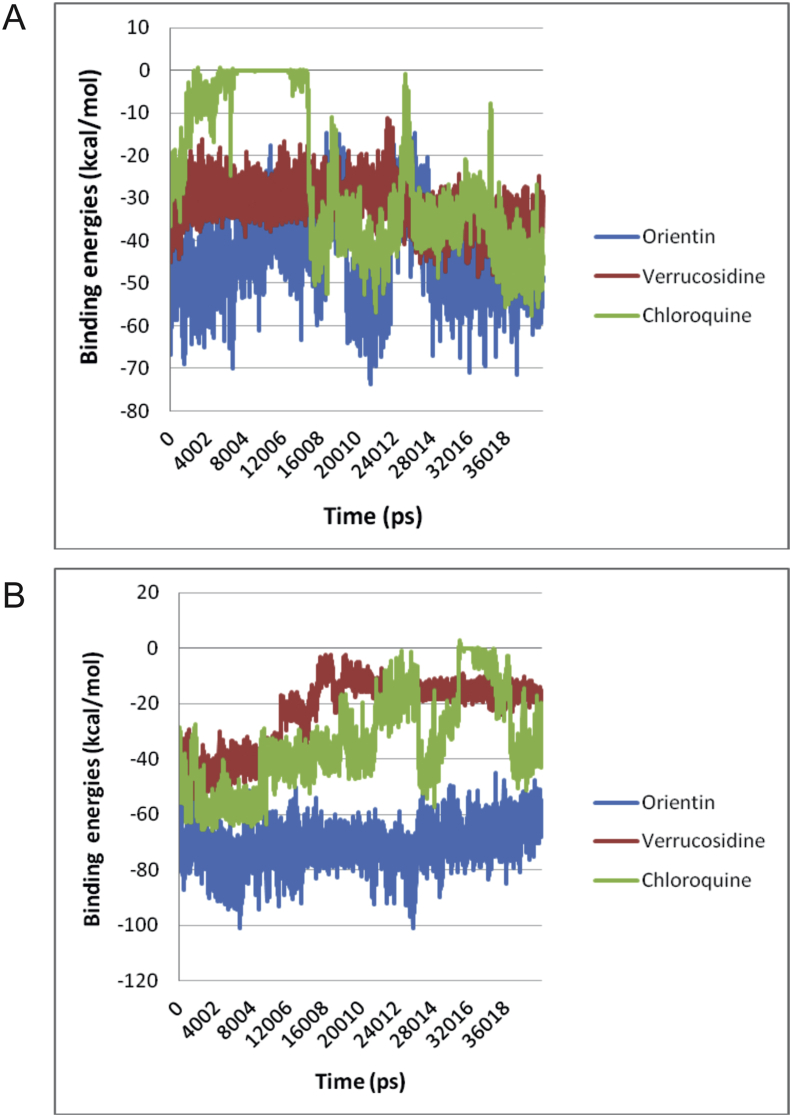
Comparison of Binding energies generated in MD simulation. Graphs represent comparison of binding energy of A) Spike protein with Orientin, Verrucosidine and Chloroquine; and B) GRP78 with Orientin, Verrucosidine and Chloroquine.

The snapshots of the complexes after 40 ns of MD simulation are shown in Figure 6D. In MD simulation, major interacting amino acid residues of GRP78 are shown in Figure 7A. Glu469, Asp552, Ala545 and Met433 are found to form hydrogen bonds with orientin whereas Lys556 forms pi-cation interaction. Hydrogen bonding, hydrophobic and water-bridges are the major interaction involved in the binding of orientin with GRP78 (Figure 7B) in MD simulation. Major interacting residues of spike receptor binding domain are shown in Figure 8A. Glu341, Asp429, Arg495, Ser336 and Asn435 are found to form hydrogen bonding with orientin. Lys333 and Try 438 form water bridges whereas Lys333 is also involved in pi-cation interaction with orientin. Hydrogen bonding, hydrophobic interactions and water bridges are also the main contributing factors in orientin spike protein binding in MD simulation (Figure 8B). Hence, based on MD simulation orientin shows good stability with protein molecules and it shows potential to inhibit the substrate binding domain of GRP78 and receptor binding region of SARS-CoV-2 spike. On the other hand, from MD simulation data (Supporting information video 5–8) binding energy of orientin is compared with binding energy of GRP78 inhibitor verrucosidin and repurposed anti-COVID-19 drug chloroquine [64]. It is observed from the result of MD simulation that orientin shows better potential of binding to both GRP78 and spike protein in comparison to verrucosidin and chloroquine (Figure 9). Average binding energies of orientin with GRP78 SBD and SARS-CoV-2 spike model as obtained from the last 10 ns of the trajectories are -66.14 ± 5.96 kcal/mol and -48.02 ± 5.49 kcal/mol, respectively, which is better than the average binding free energies of verrucosidin with RBD of SARS-CoV-2 spike protein (-36.62 ± 3.94 kcal/mol) (Figure 9A) and SBD of GRP78 (-15.98 ± 1.8 kcal/mol) (Figure 9B). Other than this, average binding energies of clinically used anti-COVID-19 drug choloquine with RBD of SARS-CoV-2 spike protein and SBD of GRP78 in last 10 ns of the trajectories are -18.73 ± 16.26 and -38.88 ± 7.74 (Figure 9), which are also not better than orientin. Hence, on the basis of *in silico* analysis orientin shows better potential of GRP78 and spike protein binding in compare to verrucosidin and chloroquine. Therefore, all these data indicate that orientin could be used to inhibit SARS-CoV-2 spike binding to GRP78, which in turn may reduce the occurrence of COVID-19.

Supplementary video related to this article can be found at https://doi.org/10.1016/j.heliyon.2021.e05923

The following is the supplementary data related to this article:

SARS-CoV-2 spike_Orientin_MD simulation_zoom SARS-CoV-2 spike_Orientin_MD simulation_zoom

GRP78_Orientin_MD simulation_zoom GRP78_Orientin_MD simulation_zoom

GRP78_Orientin_MD simulation_01 GRP78_Orientin_MD simulation_01

GRP78_Chloroquine_MD simulation GRP78_Chloroquine_MD simulation

## 4 Conclusion

SARS-CoV-2 spike model is built *in silico* from pre-existing COVID-19 spike (pdb id.-6vyb). The spike model is prepared first because of incomplete sequence and breakage within the chain of SARS-CoV-2 spike protein reported in protein data bank. This spike protein covers the whole surface of corona virus and it is the most important primary target for phytochemicals. These spikes composed of three identical chains and appear as a distinctive crown-like structure under electron microscope. Natural flavonoid orientin is found to bind *in silico* to the domain of SARS-CoV-2 spike model which is responsible for receptor binding. It is predicted that Orientin may bind to the overlapping amino acid residues of SARS-CoV-2 spike essential for SARS-CoV-2 spike – host cell receptor GRP78 binding. On the other hand, it is also observed from the data of *in silico* docking and MD simulation that orientin may also bind to the substrate binding domain of GRP78 which is essential for binding with SARS-CoV-2 spike. Overlapping binding residues of GRP78 – orientin and SARS-CoV-2 spike model – GRP78 suggests that orientin may cause inhibition of SARS-CoV-2 spike – GRP78 binding. As it is predicted that inhibition of the interaction between the COVID-19 spike protein and the host cell receptor GRP78 would possibly reduce the rate of viral infection, orientin could be an effective phytochemical to do the job. Therefore, the present *in silico* outlook suggests the possibility of using orientin as an inhibitor of SARS-CoV-2 spike protein-GRP78 binding which may pave the route for drug designers to develop suitable precautionary or therapeutic modality against COVID-19.

## Declarations

### Author contribution statement

Arijit Bhowmik, Souradeep Biswas: Conceived and designed the experiments; Performed the experiments; Analyzed and interpreted the data; Wrote the paper.

Subhadip Hajra, Prosenjit Saha: Analyzed and interpreted the data; Contributed reagents, materials, analysis tools or data; Wrote the paper.

### Funding statement

This work was financially supported by the DBT-RA Program in Biotechnology and Life Sciences, 10.13039/501100010803Department of Biotechnology, India and 10.13039/501100001411Indian Council of Medical Research.

### Data availability statement

Data will be made available on request.

### Declaration of interests statement

The authors declare no conflict of interest.

### Additional information

No additional information is available for this paper.
